# 3D‐printed microneedles with open groove channels for liquid extraction

**DOI:** 10.1002/EXP.20210109

**Published:** 2021-12-28

**Authors:** Fang Leng, Mengjia Zheng, Chenjie Xu

**Affiliations:** ^1^ Department of Biomedical Engineering City University of Hong Kong Kowloon Hong Kong SAR P. R. China

**Keywords:** 3D printing, glucose sensing, liquid extraction, microneedle

## Abstract

Microneedles (MNs) offer a simple and minimally invasive way to sample skin interstitial fluid for bioanalysis. Through the integration with portable or wearable sensing devices, it allows us to get qualitative information about some biomarkers in situ. This work is to show a MN platform with open groove channels that are manufactured using photopolymerization 3D printing. The grooves on the needle surface permit that liquid flows from the tips to the base under the influence of capillary force. The ultimate MN device can penetrate skin and tissues and sample liquid in the skin model. By taking the glucose as the model biomarker, we demonstrate that the biomarkers in the extracted liquid can be analyzed in situ by the commercial test strips attached to the back.

## Graphical abstract

This report describes the 3D fabrication of the microneedles with the open groove channels for skin interstitial fluid extraction through capillary force. Biomarkers in the extracted liquid (e.g. glucose) can be analyzed in situ by the commercial test strips attached to the back of microneedles.

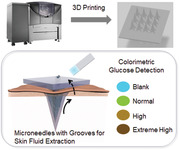

## 1 INTRODUCTION

Microneedle (MN) assisted biosensing has attracted increasing interest in recent days.^[^ ^1^ ^]^ The basic role of MNs is to sample the skin interstitial fluid (ISF) that contains both systematic and local biomarkers. The sampled ISF can be analyzed either through the conventional methods post the collection or by the portable or wearable sensing devices in situ. In comparison with conventional blood‐based diagnostics that are accurate and robust, MN‐based biosensing is less invasive, yet accurate and robust. While the emerging wearable diagnostic devices are patient‐friendly, convenient, and cost‐effective, MN‐based biosensing provides more accurate information about the biomarkers in body fluid.^[^ ^2^ ^]^

MNs currently used for skin ISF detection can be grouped into 3 categories.^[^ ^3^ ^]^ The simplest ones are the solid MNs. Although they cannot extract ISF, the interface of these devices can be engineered for directly recognizing and analyzing pre‐identified biomarkers in situ. The second type is the swellable MNs that utilize the swelling capabilities of hydrophilic polymers in skin to extract ISF. And the third type is hollow and porous MNs that rely on the capillary force to suck the ISF from the skin layers. Both the second and third types can extract skin ISF for subsequent analysis and be integrated with portable or wearable sensing devices for real‐time analysis.^[^ ^4^ ^]^ For example, we have developed the swellable methacrylated hyaluronic acid (MeHA)‐based MN patch that extracted ISF from mouse skin within minutes.^[^ ^5^ ^]^ And the glucose in the extracted ISF was quantified in situ through the electronic glucose sensors. Mishra et al. recently described a hollow MN‐based sensor array for continuous monitoring of fentanyl opioid molecule and organophosphate nerve agents. This system utilized enzymatic reaction to achieve square wave voltammetric detection of the fentanyl and nerve agent targets.^[^ ^6^ ^]^ Gu et al. developed a series of MN patches that were capable of in situ detection of hyperglycemia in mice combined with glucose biosensor.^[^ ^7^ ^]^

We notice that most MN devices for skin ISF extraction and analysis are fabricated through template molding (solid and swelling MNs) or microfabrication/micromachining (solid, hollow, and porous MNs). These methods are either simple to use or suitable for scale up. However, both methods are lack of flexibility to change or optimize the design. Fortunately, the latest development of 3D printing technology offers a solution for this limitation. For example, Krieger et al. introduced a two‐step “Print & Fill” mold fabrication method to obtain high‐aspect ratio sharp needles with tip radii of 20–40 μm.^[^ ^8^ ^]^ This was achieved using a UV‐curable resin and a desktop stereolithography apparatus 3D printer. Lim et al. printed MNs on a curved surface.^[^ ^9^ ^]^ Johnson et al. proposed the continuous liquid interface production (CLIP) to rapidly prototype MNs with tunable geometries.^[^ ^10^ ^]^ The CLIP MNs could be further dip‐coated with protein cargo.^[^ ^11^ ^]^

**FIGURE 1:**
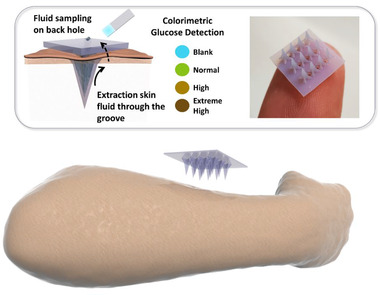
Schematic diagram of MN array with groove channels used for body fluid extraction and detection

**FIGURE 2:**
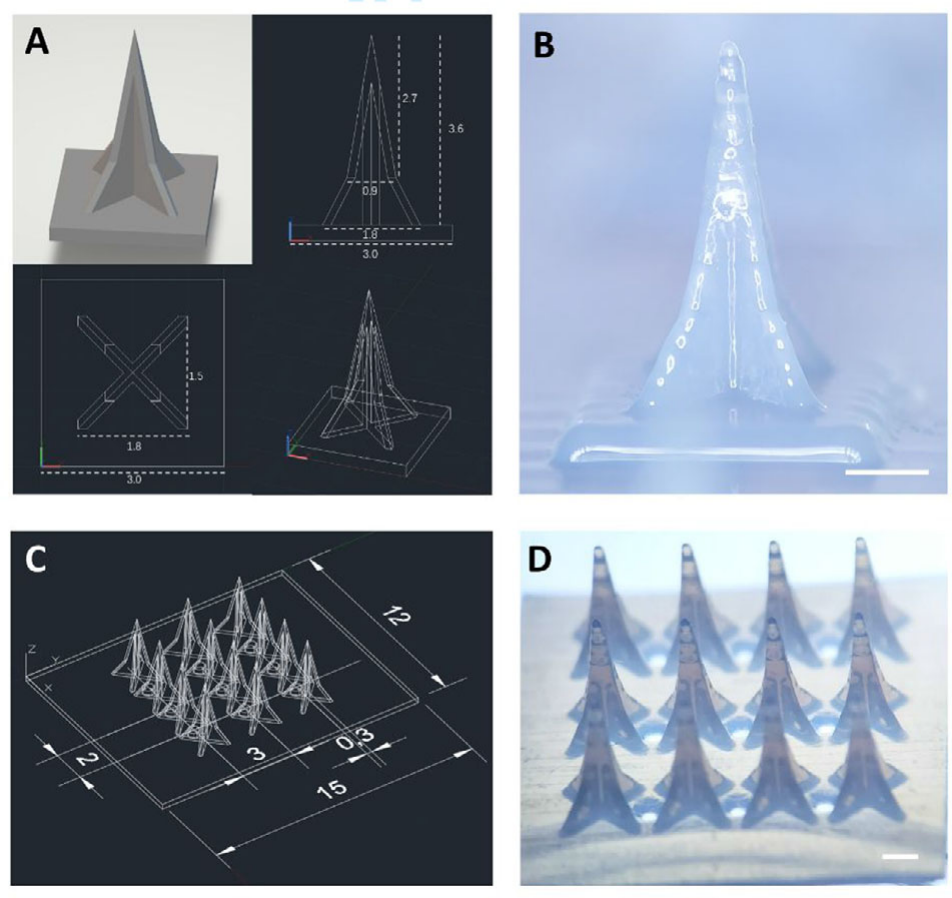
Design and fabrication of MN array. (A) CAD design of Eiffel Tower‐shaped grooved MN. (B) Microscopic image of 3D printed MN, height = 3.6 mm. (C) CAD design of 3 × 4 MN array. (D) Microscopic image of 3 × 4 MN array, 9 open channels are distributed on the plane. Scale bar = 1 mm

This paper is to show our effort in fabricating ISF extracting MNs using 3D printing (Figure 1). There are two criteria in the development. First, the resolution of 3D printing has to reach micrometers, which is determined by both printing equipment and materials. Second, the structure of MNs should allow the extraction of ISF if the MN material is not swellable. To meet the first requirement, we chose photopolymerization 3D printing technology. This method provides smooth surfaces and fine precision. It works in a similar way to inkjet document printing, jetting microscopic layers of liquid photopolymer onto a build tray and instantly cures them with UV light. The fine layers build up to create a prototype. As the finished material in photopolymerization 3D printing is non‐swellable resin, we designed the groove structures on the MN surface that extracted the liquid through capillary force (Figure 2). Compression test and penetration test showed that this MN array was strong enough to pierce into pig skin and chicken tissues. Like daggers with blood grooves, the multiple grooves provide paths to enable body fluid flow through. The grooved MN exhibited great efficiency in fluid extraction, which only took 3 s to withdraw fluid from the solution and approximately 30 s from the agarose hydrogel. Each 3 × 4 MN array allowed us to extract 20 to 30 μl of fluid without extra absorption materials. Lastly, we demonstrated its diagnostic capability by integrating a commercial glucose strip on the back of 3D printed grooved MNs, in which the integrated devices were capable to extract fluid and accurately detect the glucose levels in the hydrogel skin model.

## 2 MATERIALS AND METHODS

### 2.1 Materials preparation

Printing material (VeroBlue) and support material (SUP706) were obtained from Stratasys (USA) and used for 3D printing of MN. Glucose and sodium hydroxide were purchased from J&K Scientific (China). Commercial glucose strip (BM glucose‐test) was purchased from Sources Medicines (USA). Tissue freezing medium (Cryomatrix Clear), and adhesion slides were purchased from Epredia (China). Cryotome blade (HP35) was purchased from Thermo Fisher Scientific (USA). H&E staining kit (Ab245880) was purchased from Abcam company (UK). Phosphate buffered saline and paraformaldehyde were purchased from Sigma‐Aldrich (USA).

### 2.2 Fabrication of 3D printed MNs

All MNs were designed in AutoCAD^®^2022 software (Autodesk Inc., USA). All MNs were designed as pyramids with 4 grooves on the surface. The size of the designing objects in AutoCAD is the same as it in 3D printer, and the scale bar unit could be chosen as 1 μm, 1 mm, and 1 cm in the parameter setting. After designing processes, CAD files were then uploaded to MJ Stratasys Objet 260 Connex3 3D printer (USA). The scale bar unit was chosen as 1 mm and followed the standard processes of Objet 260 Connex3 3D printer. The printed material used for all MNs was VeroBlue resin (Stratasys Ltd., USA). The support material (SUP706, Stratasys Ltd., USA) was jetted automatically on the bottom of MNs for stabilization and overprint. After 3D printing process finished, all samples were carefully collected from the working stage. The support material was first mechanically removed, and the remaining was later removed by soaking into 3% (w/v) NaOH solution for 24 h.

### 2.3 Mechanical test of 3D printed MN arrays

The mechanical toughness of the 3D printed MN arrays was measured on Instron 5924 (Instron, Ltd. USA) with a compression test setup. The 3D printed MN patch with a 4 × 3 array was affixed on the bottom platen with needles facing upward. The initial location of the upper platen was set once it contacted with the needle tips. Later, the vertical force was applied on the MN array at a load rate of 2 mm/min until the maximum displacement of 2.0 mm was achieved.

### 2.4 Cryosection fabricating processes of tissue penetration with MN

The MN array was inserted into pig skin, chicken heart, muscle, and liver tissues by thumb press. The penetrated tissue samples were fixed in 4% paraformaldehyde for 24 h at 4 ℃. After fixation, tissue samples were rinsed and later soaked in the OCT compound. The tissue samples were frozen at −80 ℃ and sectioned on the Cryostat machine (CryoStar NX70, Thermo Fisher Scientific, Ltd. USA) with a thickness of 10 μm. The samples were stained with the Hematoxylin‐Eosin (H&E) staining following the manufacturer's protocol and examined under the microscope for penetration depth.

### 2.5 H&E staining

The slides were first hydrated in distilled water. After hydration, the slides were completely immersed in hematoxylin solution and stained for 5 min and rinsed twice in distilled water. Next, the slides were soaked in bluing reagent for 10–15 s and followed with rinsing in distilled water. Later, the tissue section slides were incubated inside the adequate Eosin Y solution for 2–3 min and then rinsed with absolute alcohol. After dehydrated with absolute ethanol, the slides were mounted with mounting media and protected with cover slip before further observation under microscope.

### 2.6 **In vitro** fluid extraction using MN arrays

The MN array was tested for liquid extraction from a solution reservoir. The array was placed parallel to the liquid surface and then inserted beneath the fluid surface for 1 mm. The liquid extraction process was filmed with camera and analyzed for the fluid extraction speed.

### 2.7 **In vitro** glucose detection on agarose hydrogel skin model

For in situ colorimetric glucose detection, the groove‐channeled MN arrays were integrated with the paper‐based glucose strips. The glucose strips were carefully cut into 0.5 cm × 0.5 cm square for each detection site and 4 detection sites were stuck on the back of each MN array. The agarose hydrogel was premixed with glucose solution to achieve the designed glucose concentrations from 0.1, 0.25, 0.5, 1, to 2 wt%. The MN arrays were inserted into the agarose gel to sample the fluid and to infiltrate the glucose strip. The detection process was filmed to analyze the required administration duration. The images of the final color were taken and compared with the color change of glucose strips directly tested with glucose solutions.

### 2.8 Statistical analysis

The statistical analysis was carried out in GraphPad Prism 8 (GraphPad Software Inc., La Jolla, CA, USA). The experimental data were reported as mean ± standard deviation (SD).

## 3 RESULTS

### 3.1 Fabrication of MNs with groove channels

Micro‐grooves utilize capillary forces to direct the flow of liquid, with no need for an extra pumping device, which makes them unique and desirable for numerous systems.^[^ ^12^ ^]^ When MN arrays with groove structure penetrate into skin, body fluid would flow through the groove channels under capillary force and accumulate on the back of MN patch.

We first explored the minimal size of MN photopolymerization 3D printing technology could offer. As shown in Figure 2A and Figure S1, we printed a series of MNs with different needle lengths, ranging from 1.44 to 9.6 mm. The groove structures were similar for all MNs. Each needle was supposed to have four grooves and four edges. However, the groove structure disappeared, and needle tip turn into blunt when the needles were smaller than 2.88 mm (Figure S1). That is mainly because the 3D printer used in this study could reach the theoretical resolution of 42 μm in *x*–*y* plane. When the height of MN was less than 2.88 mm, the average needle groove depth of upper part was shorter than 60 μm, approaching the minimal printing size. Thus, the detail structure such as the groove channel and tip cannot be fabricated for the needle with a height less than 2.88 mm.

Subsequently, we chose MNs with a height of 3.6 mm for the following experiments (Figure 2B and Figure S2). The ultimate MN patch was composed of a 3 × 4 MN array with 9 channels (Figure 2C,D) distributed on the back of patch. When MN array penetrated in the skin, body fluid could be extracted and flow through the grooves and 9 open channels to the backside of MNs. Later, it could directly collect the body fluid and detect the biomarkers by the test strips installed on the backplane.

### 3.2 Mechanical test of grooved MNs

**FIGURE 3:**
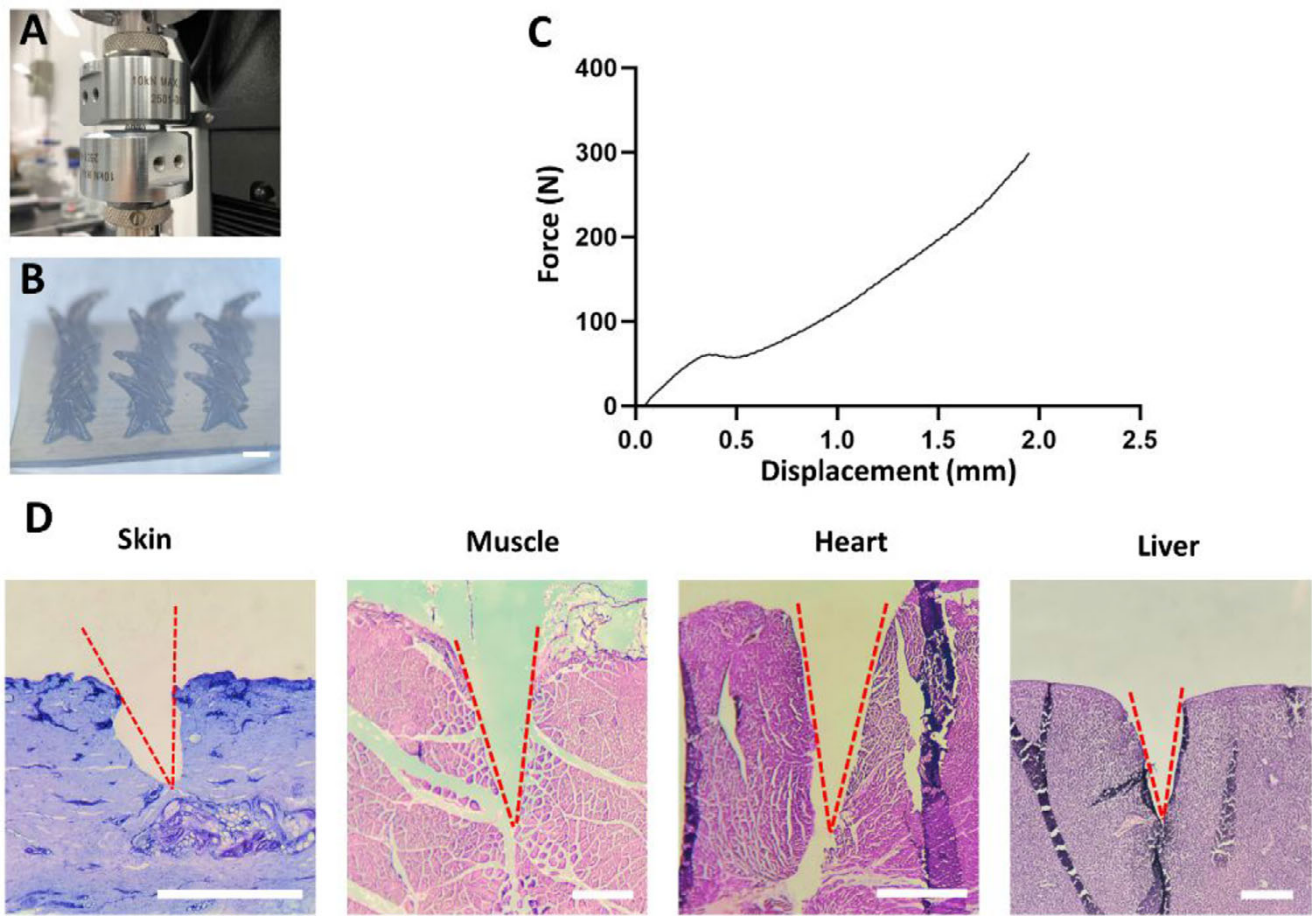
Mechanical property and tissue penetration capability of grooved MNs. (A) Compression test setup for measuring the mechanical property of grooved MN array. (B) The microscopic images of bent needles after the compression test. (C) The load‐displacement curve of the grooved MN array in the compression test. (D) H&E‐stained images of pig skin and different chicken tissues after penetrated using grooved MN arrays. The tested tissues were the pig skin, chicken muscle, chicken heart, and chicken liver, from left to right. Scale bar = 1 mm

The compression test was performed to examine the mechanical properties of printed MNs (Figure 3A). As shown in Figure 3B, all 12 MN tips bent but were not fractured after 300 N of loading. Figure 3C shows that the groove‐channeled MN array failed at 11% strain approximately, the average modulus is 6.8 MPa. We also studied their tissue penetration capabilities using pig skin and different fresh chicken tissues (muscle, heart, liver) (Figure 3D). The MNs can pierce up to a depth of 0.5 mm in pig skin and 2 mm in three different chicken tissues. The different penetrating depths between pig skin and other three tissues are mainly due to the higher toughness of pig skin. Nevertheless, these results show that the printed MNs have sufficient mechanical strength for practical usage.

### 3.3 **In vitro** fluid extraction for biomarker analysis

**FIGURE 4:**
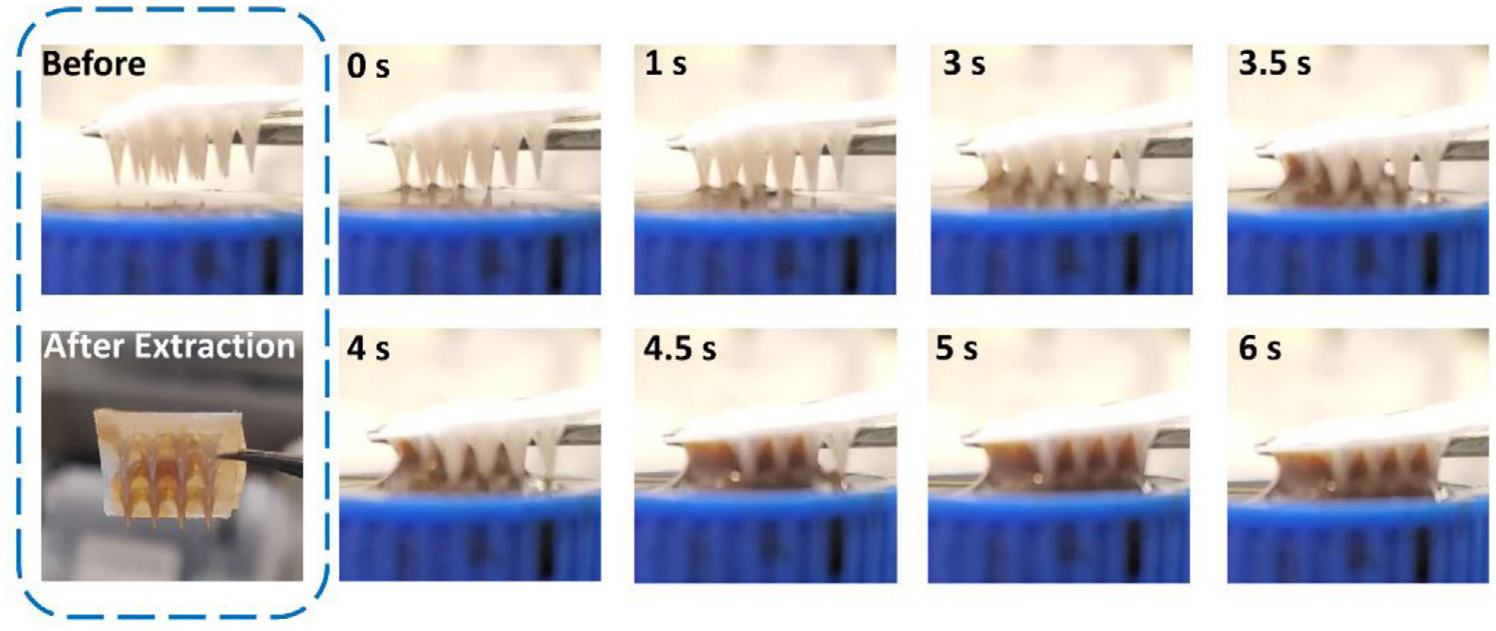
The fluid extraction behavior of the grooved MN arrays. Once contacted with liquid, the grooves of MN arrays started to extract fluid within 3 s and filled with liquid after 6 s

We evaluated the fluid extraction capability of the 3D printed MN array in the solution first (Figure 4). The MN array was placed horizontally and immersed in the liquid reservoir, while the base of MN maintained 1 mm distance from the liquid surface. Fluid started to fill the grooves only 3 s after MNs were inserted into the liquid. After 6 s, the grooves were filled with liquid. Carefully collected MN arrays and compared the weight before and after extraction, we found that each 3 × 4 MN array allowed us to extract 20 to 30 μl of fluid without extra absorption materials within 15 s (6 MN arrays were conducted the extraction test for 5 times, respectively).

**FIGURE 5:**
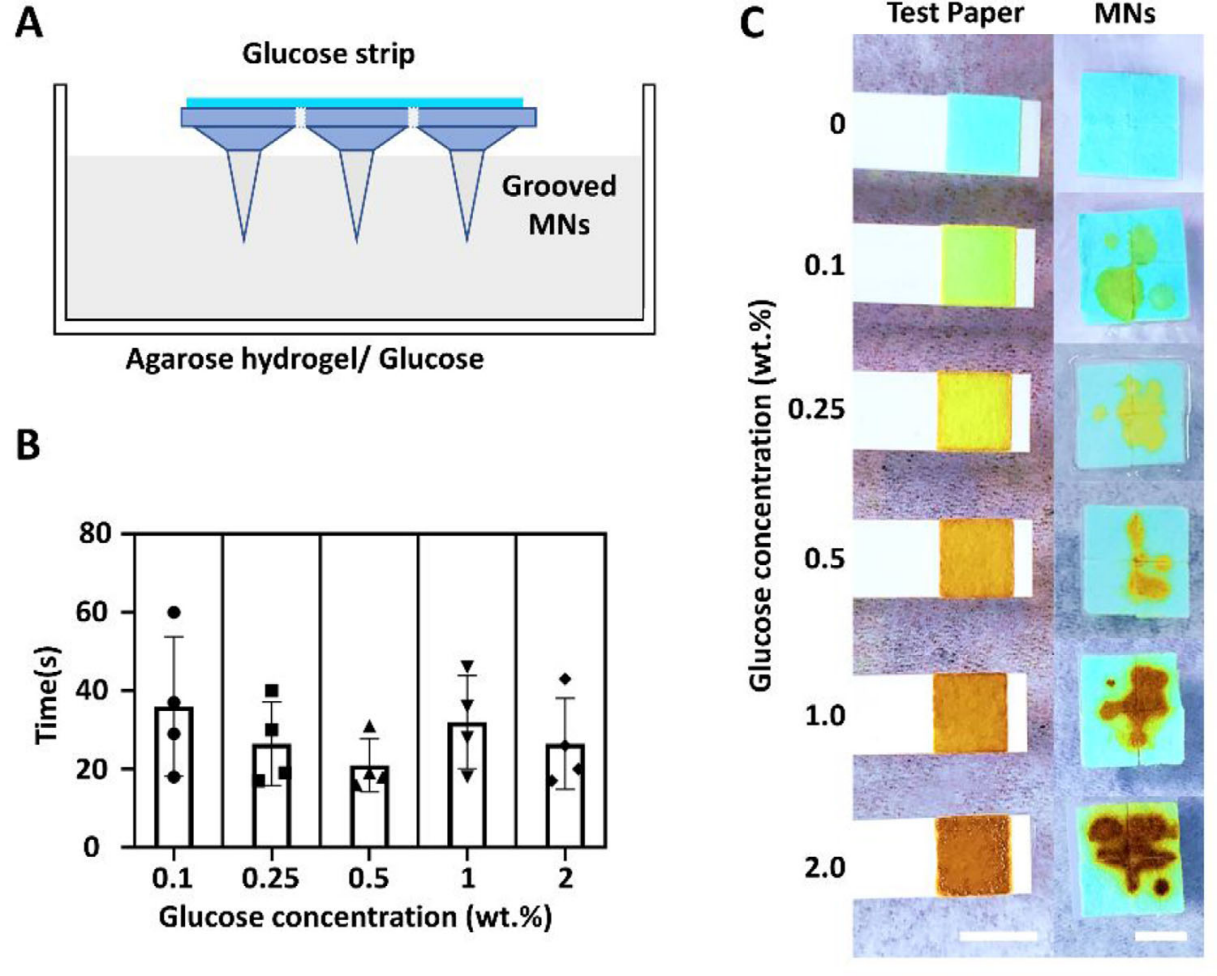
In vitro glucose detection on agarose hydrogel skin model. (A) Scheme of using grooved MNs for colorimetric glucose detection. (B) The required detection duration of the grooved MNs from 1% agarose hydrogel skin model containing different concentrations of glucose. (C) The colorimetric response of the grooved MN array on the skin phantom within 60 s application. The grooved MNs were tested on a series of agarose hydrogel containing glucose concentrations from 0.1, 0.25, 0.5, 1, to 2 wt%. The colors changes of glucose test strips in the solutions with the same glucose concentrations were compared with that obtained from grooved MNs. Scale bar = 5 mm

We further explored the possibility of biomarker detection using the extracted liquid in a hydrogel skin model (Figure 5A). This model was composed of 1% agarose hydrogel premixed with glucose to achieve a series of glucose concentrations from 0.1, 0.25, 0.5, 1, to 2 wt%. The commercial glucose strips were cut off its detection site in a size of 0.5 cm × 0.5 cm, and four detection sites were attached to the backplane of each MN array. The glucose strip modified MN array was inserted into the agarose gel with a depth of about 2 mm. The time from MN insertion to strip color stabilization is recorded. As shown in Figure 5B, the color of testing paper changed within 60 s after piercing. We compared the color change observed on the MN back with that shown on glucose strip directly dipped into the glucose solution (Figure 5C). The colorimetric responses from two groups were the same, which illustrated the integrated MN device could accurately and conveniently detect the glucose concentration from the agarose skin model.

## 4 CONCLUSIONS

This study has developed a groove‐channeled MN array with liquid extracting function. This MN array was fabricated with 3D‐printing method. In vitro extraction test showed that fluid could rapidly flow through the grooves in the vertical direction, and then be collected on the backplane for analysis. In the proof‐of‐concept, a glucose test strip was integrated into the device for analyzing the glucose concentrations in the agarose hydrogel skin model.

We notice that the 3D printing technology used here is constrained by the limited true resolution (42 μm). In the future, we will explore the use of two‐photon polymerization stereolithography which allows the fabrication of higher resolution (10 nm) structures.^[^ ^13^ ^]^ The design, material, and printing parameters will be further optimized.

## CONFLICT OF INTEREST

The authors declare no conflict of interest.

## Supporting information



SUPPORTING INFORMATION

## Data Availability

The authors declare that all data needed to support the finding of this study are presented in this article and the Supporting Information.
