# Supplementary material for: 3D‐printed microneedles with open groove channels for liquid extraction
Source: Exploration (Beijing). 2021 Dec 28;1(3):20210109. doi: 10.1002/EXP.20210109 (PMC10190842; doi:10.1002/EXP.20210109)
Supplement: Supplementary file 1 — SUPPORTING INFORMATION [file EXP2-1-20210109-s001.docx]

Supporting Information for

**3D-Printed Microneedles with Open Groove Channels for Liquid Extraction**

Fang Leng, Mengjia Zheng, Chenjie Xu ^*^

Department of Biomedical Engineering, City University of Hong Kong, Tat Chee Avenue, Kowloon, Hong Kong SAR, China

^*^ Correspondence to [chenjie.xu@cityu.edu.hk](mailto:chenjie.xu@cityu.edu.hk)


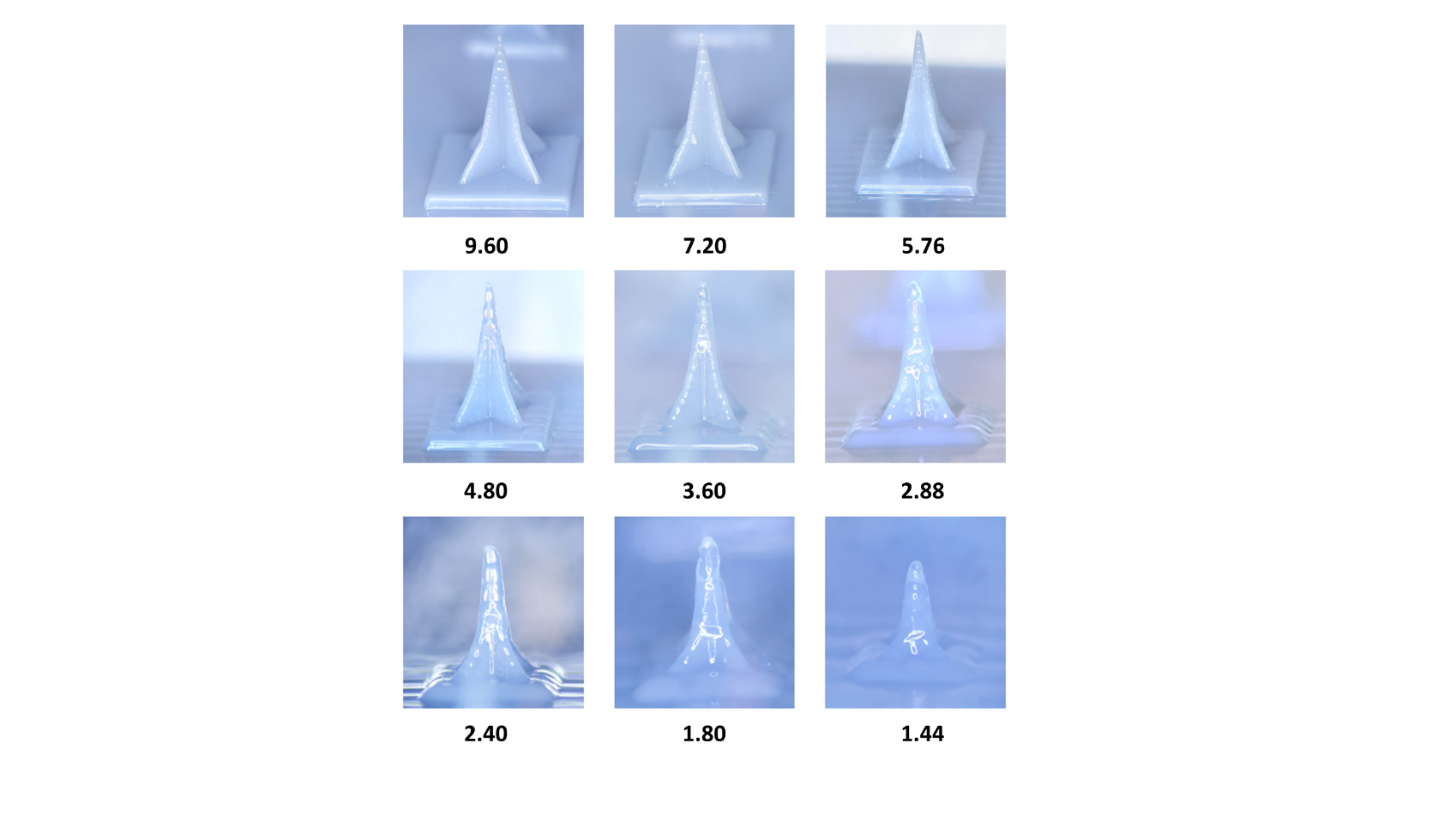


**Figure S1.** Microscopic images of Eiffel Tower inspired MN in different size (needle height= 1.44, 1.80, 2.40, 2.88, 3.60, 4.80, 5.76, 7.20, 9.60 mm).

**
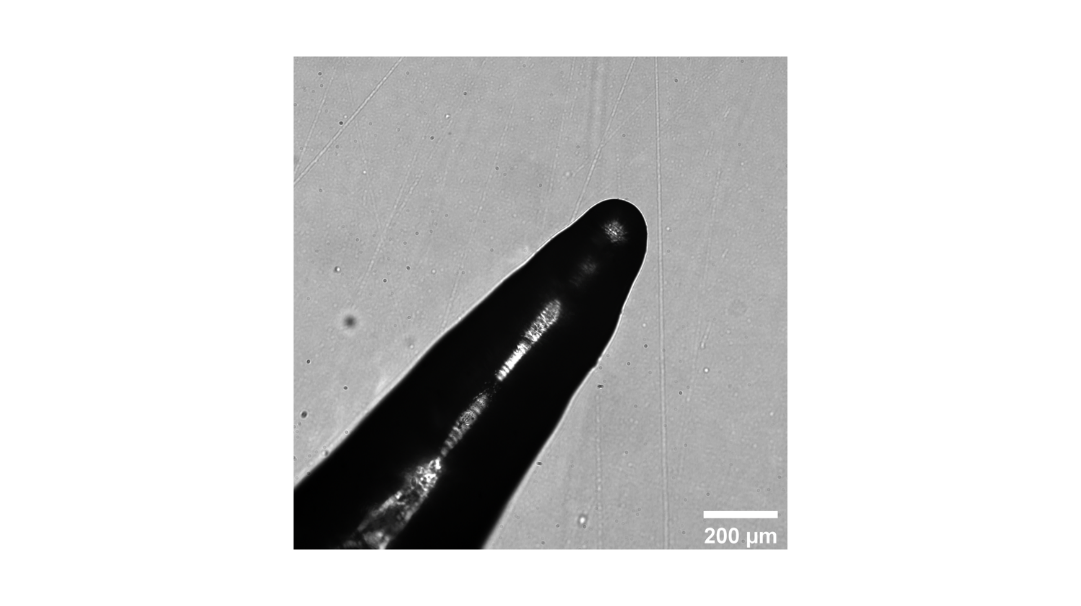
**

**Figure S2.** Microscopic image of MN tip (needle height= 3.6 mm). Scale bar= 200 μm.
